# Blood Pressure Response to Zofenopril or Irbesartan Each Combined with Hydrochlorothiazide in High-Risk Hypertensives Uncontrolled by Monotherapy: A Randomized, Double-Blind, Controlled, Parallel Group, Noninferiority Trial

**DOI:** 10.1155/2015/139465

**Published:** 2015-08-05

**Authors:** Ettore Malacco, Stefano Omboni, Gianfranco Parati

**Affiliations:** ^1^L. Sacco Hospital, 20157 Milano, Italy; ^2^Italian Institute of Telemedicine, Solbiate Arno, 21048 Varese, Italy; ^3^Istituto Auxologico Italiano and University of Milano-Bicocca, 20149 Milano, Italy

## Abstract

In this randomized, double-blind, controlled, parallel group study (ZENITH), 434 essential hypertensives with additional cardiovascular risk factors, uncontrolled by a previous monotherapy, were treated for 18 weeks with zofenopril 30 or 60 mg plus hydrochlorothiazide (HCTZ) 12.5 mg or irbesartan 150 or 300 mg plus HCTZ. Rate of office blood pressure (BP) response (zofenopril: 68% versus irbesartan: 70%; *p* = 0.778) and 24-hour BP response (zofenopril: 85% versus irbesartan: 84%; *p* = 0.781) was similar between the two treatment groups. Cardiac and renal damage was equally reduced by both treatments, whereas the rate of carotid plaque regression was significantly larger with zofenopril. In conclusion, uncontrolled monotherapy treated hypertensives effectively respond to a combination of zofenopril or irbesartan plus a thiazide diuretic, in terms of either BP response or target organ damage progression.

## 1. Introduction

Hypertension is a major modifiable risk factor for cardiovascular morbidity and mortality [1]. Effective control of high blood pressure (BP) by pharmacological treatment substantially reduces the risk of developing major cardiovascular complications, including myocardial infarction, stroke, heart failure, and kidney disease [2]. However, in most hypertensive patients and particularly in those with associated cardiovascular risk factors or at high risk for cardiovascular events, a combination therapy based on at least two drugs is required in order to achieve the recommended BP goals [3]. As a matter of fact, clinical studies and large meta-analyses have demonstrated that combination therapy allows significant improvements of both systolic BP (SBP) and diastolic BP (DBP) control in 70–80% of treated hypertensive patients [4–7], the use of combination treatment being characterized by a greater antihypertensive efficacy than the doubling of the monotherapy dose [8, 9]. For these reasons guidelines on management of hypertension currently recommend the use of two-drug combinations also as a first line therapy [3].

The association of an Angiotensin Converting Enzyme- (ACE-) inhibitor and a diuretic is amongst the preferred two-drug combinations, because the ACE-inhibitor antagonizes the counterregulatory system activity triggered by the diuretic and this results in an improvement of the efficacy and tolerability of the single drug components [3, 10].

Zofenopril calcium, a prodrug of the active compound zofenoprilat, is an ACE-inhibitor which has been successfully and safely employed in the treatment of essential hypertension [11] and acute myocardial infarction or heart failure [12], also in subgroups of patients with high BP [13, 14]. In subjects with essential hypertension zofenopril has been shown to be as effective as beta-blockers [15], diuretics [16], calcium channel blockers [17], other ACE-inhibitors [18, 19], and Angiotensin Receptor Blockers (ARBs) [20, 21].

Zofenopril has also been proved to be effective when given in combination with a thiazide diuretic [22, 23]. However, so far, only a limited fraction of subjects has been tested with the highest dose of zofenopril (60 mg) plus the diuretic, no direct comparative data on the antihypertensive efficacy and safety of the zofenopril plus hydrochlorothiazide combination versus that of an ARB plus a diuretic exist, and no information on the possible benefit of the two-drug combination on cardiovascular and renal damage is yet available. The present study was planned and conducted in order to bridge this void, by selecting essential hypertensive patients not controlled by a previous monotherapy, and with one or more additional cardiovascular risk factors.

## 2. Methods

### 2.1. Study Population

Essential hypertensive subjects of both genders, aged 18 to 75 years, with at least one additional cardiovascular risk factor, and taking one antihypertensive medication among ACE-inhibitors, ARBs, calcium channel blockers, diuretics, and beta-blockers, in the last 3 months, but not adequately controlled (sitting office SBP ≥140 mmHg and/or sitting office DBP ≥90 mmHg), were eligible for study participation. The following cardiovascular risk factors were considered among the inclusion criteria [3]: (a) current smoking; (b) elevated total cholesterol (>190 mg/dL) or specific lipid-lowering drug treatment; (c) elevated Low Density Lipoprotein (LDL) cholesterol (>115 mg/dL) or specific lipid-lowering drug treatment; (d) low High Density Lipoprotein (HDL) cholesterol (<40 mg/dL in males and <46 mg/dL in females) or specific lipid-lowering drug treatment; (e) fasting plasma glucose between 102 and 125 mg/dL or being on specific drug treatment for hyperglycemia; (f) diabetes mellitus (fasting plasma glucose ≥126 mg/dL) controlled by diet or specific antidiabetic therapy; (g) abdominal obesity: waist circumference ≥102 cm in males and ≥88 cm in females, or Body Mass Index (BMI) between 25 and 29.9 kg/m^2^; and (h) family history of premature cardiovascular disease (males at age <55 years and females at age <65 years).

Patients were excluded if they had (a) secondary or malignant hypertension; (b) orthostatic hypotension (office SBP drop upon standing ≥20 mmHg); (c) history of heart failure requiring medical treatment; (d) myocardial infarction or cerebrovascular accidents in the previous 6 months; (e) hemodynamically significant cardiac valve disease; (f) severe or clinically significant systemic, renal, hepatic, neurological, or psychiatric disease; (g) obesity (BMI ≥30 kg/m^2^); (h) large (circumference >32 cm) or tiny upper arm (circumference <24 cm); (i) known hypersensitivity to ACE-inhibitors, ARBs, or thiazide diuretics.

Pregnant women and breastfeeding mothers were excluded as well. Premenopausal women with childbearing potential had to practice an effective method of birth control and were required to have a negative urine pregnancy test.

The study was conducted according to Good Clinical Practice guidelines and the Declaration of Helsinki, and the protocol was approved by the ethics committees of the centers involved. Written informed consent was obtained from all patients prior to their inclusion into the study.

### 2.2. Study Design

This was an Italian, multicenter, randomized, double-blind, parallel group study, conducted at 34 Italian hospitals. The first patient was enrolled in May 2009 and the last patient was enrolled in January 2012. Patients were randomized 1 : 1, using a centralized, computer-generated randomization list in blocks of 4. The study consisted of a 2-week run-in period during which current monotherapy was continued unchanged, followed by 18 weeks of double-blind treatment with zofenopril or irbesartan at the initial doses of 30 and 150 mg combined with hydrochlorothiazide 12.5 mg. Study drugs were given orally and once daily (between 9 and 11 a.m.) with a glass of water. The two study treatments were supplied as identical oral tablets (overencapsulation technique). After the first 6 and 12 weeks of active treatment the dose of zofenopril and irbesartan had to be doubled, respectively, to 60 and 300 mg, if office SBP was ≥140 mmHg or office DBP was ≥90 mmHg in nondiabetic patients and if office SBP was ≥130 mmHg or office DBP was ≥80 mmHg in diabetic patients or in patients with at least 3 cardiovascular risk factors.

At the screening visit informed consent was obtained, medical history was collected, and a physical examination, BP and heart rate measurements, and laboratory tests (blood count, glucose, total, LDL, and HDL cholesterol, triglycerides, uric acid, creatinine, sodium and potassium, transaminases and *γ*-GT, total bilirubin, urinalysis, and urine pregnancy test) were assessed. At randomization visit, 2 weeks after inclusion, a 12-lead ECG, an echocardiogram, and a carotid ultrasonography were carried out. Physical examination and BP and heart rate measurements were repeated at each visit (6, 12, and 18 weeks after randomization), while an ECG, an echocardiogram, and a carotid ultrasonography were assessed again and laboratory tests (including urine pregnancy test) were rechecked at the end of the 18 weeks of double-blind treatment. Adverse events, use of concomitant medications, and compliance with treatment were assessed at each visit. At the end of the 2-week run-in period and of the 18 weeks of double-blind treatment BP was also measured by 24-hour ambulatory monitoring.

The study also included a 30-week double-blind follow-up period for patients uptitrated to the high drug dose of zofenopril or irbesartan during the first 18 weeks. During this period patients were seen initially after 6 weeks and then every 8 weeks: office BP was measured at each visit, whereas a 24-hour ambulatory BP monitoring was performed at the end of the 30 weeks.

### 2.3. Office BP and Heart Rate Measurement

BP and heart rate were measured in the office by a validated, automatic, electronic, upper-arm sphygmomanometer (A&D UA-767PC, A&D Company Limited, Tokyo, Japan) [24], approximately 24 hours after the last placebo or drug intake. The arm cuff was kept at the heart level during every BP measurement. Three measurements, taken at 2 min intervals, after 5 min of rest in the sitting position were averaged and used as the office BP reference value. BP and heart rate values were taken also after 1 and 4 minutes of standing.

### 2.4. Ambulatory BP Measurements

Ambulatory BP monitoring was performed at randomization and the final visit, noninvasively over the 24 hours by an oscillometric, validated, automatic, electronic device (A&D TM-2430, A&D Company Limited, Tokyo, Japan) [25]. The monitoring cuff was wrapped around the nondominant arm and the patient was asked to keep her/his arm still during the automatic BP measurements. The device was programmed to provide automatic readings every 15 min throughout the whole monitoring period. Each recording started in the morning, immediately after office BP assessment and administration of placebo or active treatment. Patients were then sent home and asked to come back 24 hours later. They were instructed to attend their usual activities during the monitoring period, avoiding strenuous exercise, and to keep the arm extended and immobile during the automatic cuff inflations. Results of the recording were read by connecting the BP measuring device to a wireless interface which sent data to a centralized data management center through the mobile telephone network and the web [26]. Traces had to be analyzed in real time and in case of a bad quality recording (see below) the investigator was contacted in order to repeat the recording in the next two days, whenever possible.

### 2.5. Echocardiography

In order to obtain reliable and accurate tests, echocardiograms were performed by operators trained and certified during a course held at the coordinating center before study initiation. Echocardiograms were obtained with the subject in left lateral decubitus, after 30 minutes of rest. Only one certified physician in each center was responsible for recording the echocardiograms. Left ventricular internal diameters, left ventricular posterior wall thickness, and interventricular septum thickness were measured monodimensionally from the longitudinal parasternal view previously identified bidimensionally, according to the indications of the American Society of Echocardiography [27]. Left ventricular mass was calculated according to the Penn Method [28] and indexed to body surface area by using the formula of D. Du Bois and E. F. Du Bois [29]. Left ventricular hypertrophy was considered to be present if the left ventricular mass index (LVMI) exceeded 125 g/m^2^ in males or 110 g/m^2^ in females [3]. Since LVMIs were calculated locally only by few centers and using different algorithms, they were recalculated centrally and blindly from the original measures, before the database lock.

### 2.6. Carotid Ultrasonography

Also in case of carotid ultrasonography, in order to obtain reliable and accurate reports, the test was performed by operators trained and certified during a course held at the coordinating center prior to study initiation. Patients were examined in the supine positions by B-mode carotid scans. The carotid arteries were interrogated using an ultrasound system with a linear-array transducer operating at a fundamental frequency of at least 7 MHz. Examinations of carotid artery and measurement of intima-media thickness (IMT) were obtained manually at different carotid sites (common, bifurcation, external, and internal carotid artery), from at least 3 different angles of incidence in each segment, as recommended by current guidelines [30]. The three measurements taken at each segment were averaged and the average was used as the reference for each segment. Ultrasound scans were performed by only one certified sonographer at each referral center and were read locally. Data were expressed as the maximum IMT at each carotid segment explored. Atherosclerotic plaque was defined as an IMT >1.3 mm in any of the segments examined [30, 31]. The choice of 1.3 mm was done because at the time the protocol was devised and implemented on field this was the threshold recommended by guidelines and this was used as a reference for patients' management by the investigators at each study site [31].

### 2.7. Data Analysis

The primary efficacy study end-point was the intertreatment comparison in the rate of office sitting BP response (<140/90 mmHg in nondiabetics and <130/80 mmHg in diabetics or high-risk patients, or SBP reduction ≥20 mmHg or DBP reduction ≥10 mmHg) at the end of the 18 weeks of double-blind treatment.

This was a noninferiority trial and thus the hypothesis was that zofenopril plus hydrochlorothiazide and irbesartan plus hydrochlorothiazide had to be defined as equivalent in case of a difference <10% in the rate of responders after 18 weeks of treatment. This choice was based on previous evidence on response rate in zofenopril- and irbesartan-based studies [20, 21, 32, 33], and its appropriateness has been documented in a twin study, recently published [34]. Using a one-sided two-group large-sample normal approximation test of proportions at the 2.5% level, with a power of 80%, the estimated minimum number of patients to be randomized was 446 (including a 10% drop-out rate), 223 for each treatment group.

Analysis was performed on patients valid for intention-to-treat analysis, defined as all randomized patients receiving at least one dose of active treatment drug and having at least one office BP measurement after randomization. The last observation carried forward method was used for patients prematurely leaving the study. The per-protocol population included all randomized patients completing the 18-week double-blind study period without major protocol violations and was used for confirmatory analysis.

Secondary study end-points included intertreatment comparison of (a) changes in sitting office SBP and DBP after 18 weeks of treatment; (b) percentage of patients with an average 24-hour BP <130/80 mmHg or a SBP reduction ≥10 mmHg or a DBP reduction ≥5 mmHg at study end; (c) changes in LVMI with treatment and percentage of patients with cardiac damage at study end (LVMI ≥125 g/m^2^ in males or ≥110 g/m^2^ in females) [3]; (d) changes in albumin-creatinine ratio and microalbuminuria with treatment and percentage of patients with renal damage at study end (albumin-creatinine ratio ≥22 mg/g for males and ≥31 mg/g for females or microalbuminuria, evaluated quantitatively or semiquantitatively by dipstick, between 30 and 300 mg/24 h) [3]; (e) changes in maximum IMT with treatment and percentage of patients with vascular damage at study end (maximum IMT >1.3 mm in any district) [30, 31]. Rate of office BP responders, office and 24-hour SBP and DBP changes from baseline, and changes in cardiac, renal, and vascular damage from baseline at week 48 were the study end-points for the double-blind extension phase.

The analysis of 24-hour BP recordings was preceded by removal of artifacts according to previously described editing criteria [35]. Recordings were considered valid when no more than 1 hour was missing over the 24 hours and when at least 70% of expected measurements were available.

For the primary study end-point and for the changes in sitting office SBP and DBP a subgroup analysis was also done, considering subjects taking the low dose of both drugs (zofenopril 30 mg and irbesartan 150 mg). For the primary end-point, this subgroup analysis was applied to all subjects and also to those with mild (office SBP 140–159 mmHg and DBP 90–99 mmHg) and moderate-severe hypertension (office SBP ≥160 mmHg and DBP ≥100 mmHg).

Safety analysis was applied to all randomized patients, by calculating the incidence of adverse events and changes in laboratory data or ECG during the study.

Intertreatment differences for the primary study end-point were tested using a chi-square test, correcting by the center effect: the 95% confidence interval of the difference in proportion was calculated and the lower bound was compared with the 10% noninferiority limit. The same analysis was applied to secondary end-points with a discrete distribution, while differences between the two randomization groups for continuous variables were tested by analysis of variance, or, in the case of target organ damage measures, by analysis of covariance (continuous variables) or logistic regression analysis (discrete variables), by adjusting for the baseline value and other potentially confounding variables (age, gender, abdominal obesity, HDL cholesterol, family history for premature cardiovascular disease, baseline BP, and BP changes with treatment). The rates of patients experiencing an adverse event were compared between the two treatment groups by a logistic regression analysis, taking into account treatment and confounding variables. The level of statistical significance was kept at 0.05 throughout the whole study. Data are shown as mean ±SD, as mean and 95% confidence interval, and as absolute (*n*) or relative (%) frequency.

## 3. Results

### 3.1. Baseline Demographic and Clinical Data

**Figure 1:**
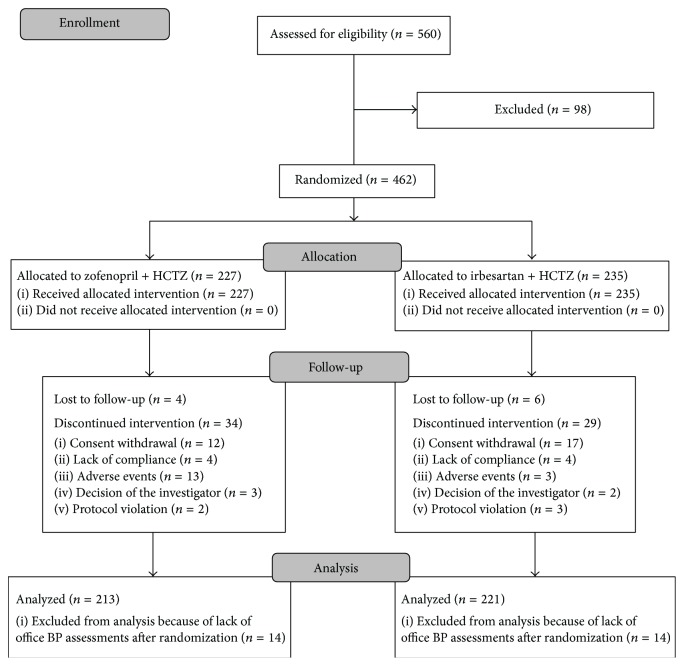
Flow diagram of the patients through the different phases of the study.

A total of 560 patients were screened, but 98 were lost during the run-in period. Thus the number of patients randomized to one of the two treatment arms was 462: 389 of these patients completed the 18-week double-blind randomized phase, while 73 discontinued the study because of consent withdrawal (*n* = 29), adverse events (*n* = 16), loss to follow-up (*n* = 10), lack of compliance with study procedures (*n* = 8), decision of the investigator (*n* = 5), or protocol violation (*n* = 5). A flow diagram of the patients throughout the study is presented in Figure 1.

Overall 434 patients were valid for the intention-to-treat analysis (213 in the zofenopril plus hydrochlorothiazide and 221 in the irbesartan plus hydrochlorothiazide treatment group) and 302 for the per-protocol analysis (146 in the zofenopril plus hydrochlorothiazide and 156 in the irbesartan plus hydrochlorothiazide treatment group). 229 out of 438 patients undergoing ambulatory BP monitoring at baseline had valid recordings and were included in this subgroup analysis (113 randomized to zofenopril plus hydrochlorothiazide and 116 to irbesartan plus hydrochlorothiazide).

A total of 244 patients, uptitrated to the high drug dose of zofenopril (*n* = 130) or irbesartan (*n* = 114), entered the double-blind extension phase and were followed for 30 weeks; 223 of them were included in the intention-to-treat analysis for this period (119 in the zofenopril plus hydrochlorothiazide and 104 in the irbesartan plus hydrochlorothiazide treatment group). The number of patients with valid ambulatory BP recordings at the end of the extension phase was 50 in the zofenopril and 44 in the irbesartan group.

As shown in Table 1, patients randomized to zofenopril plus hydrochlorothiazide were homogeneous for baseline demographic and clinical characteristics. This was the case also for the subgroup of patients included in the ambulatory BP monitoring analysis (data not shown). Most of the enrolled subjects had at least 3 major cardiovascular risk factors and were thus classified as being at high or very high cardiovascular risk.

### 3.2. Drug Dosing

The full dose of zofenopril (60 mg) in combination with hydrochlorothiazide was taken at the end of the study by 55.9% of patients randomized to this drug, while the full dose of irbesartan (300 mg) was taken by 47.1% of patients (*p* = 0.066).

### 3.3. Sitting Office BP Responders

**Figure 2:**
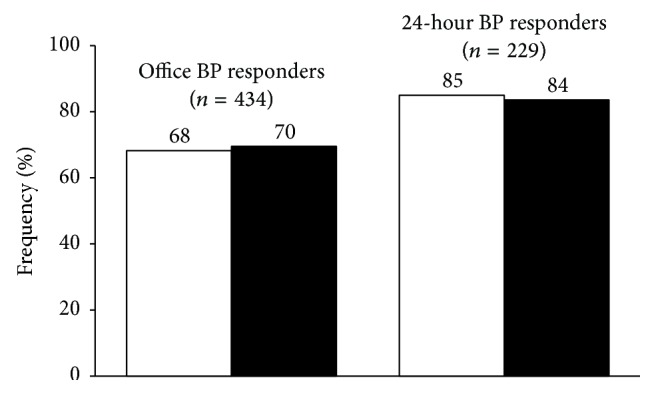
Percentage (%) of office blood pressure (BP) responders (<140/90 mmHg in nondiabetics and <130/80 mmHg in diabetics or high-risk patients, or SBP reduction ≥20 mmHg or DBP reduction ≥10 mm) and of 24-hour BP responders (<130/80 mmHg or a SBP reduction ≥10 mmHg or a DBP reduction ≥5 mmHg) after 18 weeks of treatment with zofenopril 30–60 mg plus hydrochlorothiazide 12.5 mg (open bars) and irbesartan 150–300 mg plus hydrochlorothiazide 12.5 mg (full bars). Data are shown for the intention-to-treat population (*n* = 434 office BP set; *n* = 229 ambulatory BP set).

As shown in Figure 2, the primary study end-point (proportion of patients with office BP <140/90 mmHg in case of nondiabetics and <130/80 mmHg in case of diabetics or high-risk patients, or SBP reduction ≥20 mmHg or DBP reduction ≥10 mmHg) was achieved by the end of the 18 weeks of double-blind treatment by a similar proportion of patients treated with zofenopril plus hydrochlorothiazide (68.2%) and irbesartan plus hydrochlorothiazide (69.5%, *p* = 0.778). The odds ratio for the difference in the response to treatment (zofenopril plus hydrochlorothiazide versus irbesartan plus hydrochlorothiazide) was 1.066 (95% confidence interval: 0.685–1.659): since the observed lower bound of the confidence interval (0.685) was higher than that estimated for a 10% difference (0.580), the achievement of the noninferiority criterion could be confirmed.

In the patients taking the low drug doses at study end, the proportion of responders did not differ (*p* = 0.693) between zofenopril (76.4%) and irbesartan (78.9%). The odds ratio for treatment differences was estimated at 1.159 (0.456, 2.415): as for the whole study group and also for the low dose subgroup the observed lower bound of the confidence interval (0.456) was higher than that estimated for a 10% difference (0.420), and thus the noninferiority assumption could be satisfied.

The rate of responders for the two low dose drug treatments was similar also in subgroups of patients with mild hypertension (74.6% zofenopril versus 78.8% irbesartan; odds ratio and 95% confidence interval: 1.267 (0.587, 2.736), *p* = 0.546) and moderate hypertension (88.9% versus 80.0%; 0.500 (0.037, 6.683), *p* = 0.596).

### 3.4. Sitting Office BP Changes

**Figure 3:**
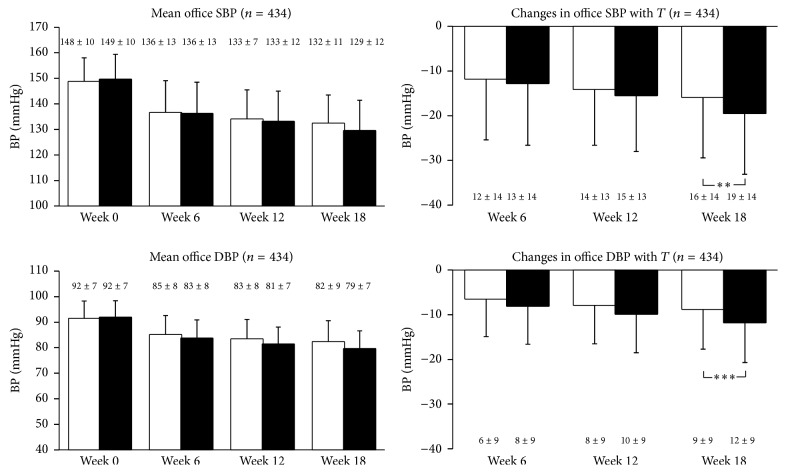
Mean office systolic blood pressure (SBP) and diastolic blood pressure (DBP) values and changes with treatment (*T*) in patients treated with zofenopril 30–60 mg plus hydrochlorothiazide 12.5 mg (open bars) or irbesartan 150–300 mg plus hydrochlorothiazide 12.5 mg (full bars). Data are shown for the intention-to-treat population and as mean values ±SD. Asterisks refer to the statistical significance of the intertreatment differences (^*∗∗*^ *p* < 0.01; ^*∗∗∗*^ *p* < 0.001).

Sitting office BP values were progressively and significantly (*p* < 0.01) reduced by both treatment regimens during the study (Figure 3). At the final evaluation (week 18), sitting office SBP and DBP were reduced on average (±SD) by 15.7 ± 13.7 and 8.6 ± 9.1 mmHg with zofenopril combined with the diuretic and by 19.3 ± 13.8 and 11.6 ± 9.1 mmHg with irbesartan + hydrochlorothiazide (intertreatment differences: *p* < 0.01 for SBP and *p* < 0.001 for DBP) (Figure 3 and Table 2). In patients taking the low drug dose SBP and DBP reductions were similar between the two treatment groups (Table 2).

### 3.5. 24-Hour Ambulatory BP Responders

The percentage of responders over the 24 hours (average 24-hour BP <130/80 mmHg or SBP reduction ≥10 mmHg or a DBP reduction ≥5 mmHg at study end) was higher than that of office responders and similar for zofenopril- and irbesartan-treated patients (85.0 versus 83.6%, *p* = 0.781) (Figure 2).

Both treatments effectively reduced ambulatory BP. 24-hour SBP was similarly (*p* = 0.050) reduced by zofenopril + hydrochlorothiazide (from 129.4 ± 10.4 to 122.7 ± 9.4 mmHg; reduction of 6.4 (8.4, 4.4) mmHg) and irbesartan + hydrochlorothiazide (from 131.8 ± 11.8 to 120.9 ± 10.9 mmHg, 9.2 (11.1, 7.3) mmHg), while for 24-hour DBP significantly (*p* = 0.001) higher reductions were obtained under the irbesartan + hydrochlorothiazide (from 80.2 ± 8.1 to 73.0 ± 6.8 mmHg, reduction of 7.0 (8.2, 5.7) mmHg) than under the zofenopril + hydrochlorothiazide combination (from 81.4 ± 8.1 to 76.8 ± 6.9 mmHg, 3.6 (4.9, 2.3) mmHg).

We did not perform an analysis for 24-hour BPs on the low dose subgroup, since the sample size (34 patients receiving zofenopril and 49 receiving irbesartan) was too small to obtain reliable results.

### 3.6. Treatment Effect on Target Organ Damage

A summary of the effects of treatment on target organ damage measures is reported in Table 3. Both treatments had a similar positive effect on regression of cardiac and renal damage. A small reduction in the maximal IMT detected at any examined carotid district was also observed, with a significantly (*p* = 0.047) larger chance of carotid plaque regression under zofenopril (31.6%) than under irbesartan (16.1%).

### 3.7. Double-Blind Extension Period

For the 223 patients receiving drug dose uptitration at the end of the 18 weeks of treatment and continuing the study for additional 30 weeks, no differences were observed in office BP response and office and 24-hour BP reductions between the two treatment groups (Table 4). Similarly, the impact of treatment on target organ damage did not significantly differ between the two study drugs (Table 5).

### 3.8. Safety and Tolerability

Laboratory and safety analyses were carried out in all randomized patients (*n* = 462). A total number of 62 (13.4%) patients (37 in the zofenopril plus hydrochlorothiazide and 25 in the irbesartan plus hydrochlorothiazide treatment group, *p* = 0.075) reported 73 adverse events (43 under zofenopril and 30 under irbesartan). Most of the adverse events (69.9%) were of a mild intensity. Eighteen (3.9%) patients were withdrawn from the study due to adverse events, 14 in the zofenopril and 4 in the irbesartan treatment group; of these patients, 9 (4.0%) receiving zofenopril and 3 receiving irbesartan (1.3%) reported drug-related adverse events (*p* = 0.091).

A total of 29 drug-related adverse events (22 under zofenopril and 7 under irbesartan) occurred in 24 patients (5.1%), of which 17 (7.5%) were treated with zofenopril plus the diuretic and 7 (3.0%) with irbesartan plus the diuretic (*p* = 0.052). The most common drug-related adverse event observed under zofenopril was cough (4 cases), whereas erectile dysfunction was the most prevalent drug adverse reaction in irbesartan-treated patients (2 cases).

In the extension phase 16 patients under high dose zofenopril plus hydrochlorothiazide (12.3%) and 13 patients under high dose irbesartan plus hydrochlorothiazide (11.4%) reported an adverse event (*p* = 0.843). Treatment-related adverse events occurred in 5 (3.8%) and 4 (3.5%) patients under the two study drugs (*p* = 0.859): of these patients, 4 were definitely withdrawn from the extension phase (3 zofenopril versus 1 irbesartan, *p* = 0.368).

Treatment was accompanied by either no change or only small and meaningless changes in the laboratory values considered in the study.

## 4. Discussion

Our study aimed at comparing the efficacy of 18 weeks of treatment with zofenopril 30 or 60 mg plus hydrochlorothiazide 12.5 mg once daily and irbesartan 150 or 300 mg plus hydrochlorothiazide 12.5 mg once daily in hypertensive patients uncontrolled by a previous monotherapy and with additional cardiovascular risk factors. At the end of follow-up the proportion of responders to treatment (<140/90 mmHg in nondiabetics and <130/80 mmHg in diabetics or high-risk patients, or SBP reduction ≥20 mmHg or DBP reduction ≥10 mmHg) was similar in zofenopril- and irbesartan-treated patients (68 versus 70%), with a similar use of the highest drug dose (56% zofenopril versus 47% irbesartan). The good office BP control obtained with either drug was confirmed over the 24 hours by ambulatory monitoring. At study end, both drugs yielded a similarly high percentage of 24-hour responders (<130/80 mmHg or a SBP reduction ≥10 mmHg or a DBP reduction ≥5 mmHg): 85% zofenopril versus 84% irbesartan.

The large proportion of responders in both treatment arms supports the finding of previous studies that, in most patients not responding to a single antihypertensive medication, combination treatment with two drugs may substantially increase the chance of response [8, 9]. Our results also confirm that combination treatment between a drug acting on the angiotensin-renin-aldosterone system and a thiazide diuretic should be among the preferred choices when monotherapies fail to lower BP to or below target levels [36].

It is worth noticing that more than 90% of our subjects displayed multiple risk factors, which placed them in the high- or very high-risk category [3]. In these subjects current guidelines recommend initiation with a low dose two-drug combination, which may offer the advantage of a prompter response and a greater probability of achieving the target BP [3]. As a matter of fact in the relatively small subset of patients taking the low drug dose at study end (30 mg for zofenopril and 150 mg for irbesartan) the rate of responders was high and did not significantly differ between the two groups (76% versus 79%). This means that even the low dose combination treatment has a high chance of success in patients previously classified as nonresponders to monotherapy and thus might represent a reasonable initial approach for treating these patients. This also strengthens the evidence from previous large randomized studies in patients with mild-moderate hypertension, in which treatment with the low dose of zofenopril (30 mg) combined with hydrochlorothiazide 12.5 mg once daily showed a greater efficacy than the monotherapy with either agent, with an increase in the response rate up to 55–65% [22, 23].

We also explored the effect of both treatments on target organ damage but could not find any relevant difference between the two study drugs. Likely, this was due to the fact that the study was not designed to assess this objective and the study sample was not adequate to detect a clinically plausible difference. A specific, adequately designed study in a large sample of patients and over a longer follow-up period might address this aspect.

This is the first study assessing the antihypertensive efficacy of high dose zofenopril (60 mg) plus hydrochlorothiazide 12.5 mg. In a previous dose-finding multifactorial study after 12 weeks of treatment with zofenopril 30 or 60 mg plus hydrochlorothiazide 12.5 mg the overall proportion of DBP responders to zofenopril plus hydrochlorothiazide was 86% and that of SBP responders was 60% [32]. As far as the benefits of the irbesartan and hydrochlorothiazide combination therapy are regarded, these have been documented in a number of trials in patients with mild hypertension, including those failing to achieve BP control with monotherapy, showing normalization rates up to 80–85% [37–40].

This is also the first study comparing zofenopril in combination with a thiazide diuretic with an ARB combined with a diuretic: previous direct comparative studies based on zofenopril monotherapy did not show any relevant difference in treatment efficacy versus an ARB-based monotherapy regimen [20, 21]. The combination between irbesartan and a thiazide diuretic has also never been directly compared against that of an ACE-inhibitor plus a diuretic, while evidence from comparative trials versus an ACE-inhibitor monotherapy is available, showing better BP response than enalapril [41–44] and fosinopril [45].

Both combination treatments were well tolerated, with a very limited number of drug-related adverse events. As expected, the combination containing zofenopril was associated with more cases of cough (6 versus none with irbesartan), but only for one patient cough led to study drug discontinuation. Other adverse drug reactions were well balanced between the two groups and the overall tolerability profile of zofenopril and irbesartan, including metabolic adverse effects, was comparable with that of previous reports [22, 46, 47].

## 5. Study Limitations

Finally, some limitations of the present study deserve to be discussed. The number of subjects included in the intention-to-treat analysis halved when the subgroup of patients undergoing ambulatory BP monitoring was considered. This occurred because many recordings were missing or qualitatively inadequate. However, in the ambulatory BP monitoring subgroup the percentage of responders was still comparable between the two treatment groups, indicating that the two populations were homogeneous. Evaluation of hypertension organ damage was done locally at each site and no centralized reading or specific quality control of original examinations (including ultrasound scans, serum creatinine, and urinary albumin) was planned. The fact that the measurements were done manually makes it highly unlikely to have enough sensitivity and reproducibility to detect reliable intertreatment changes in the short period of follow-up, particularly for segments such as the internal and external carotid artery which are very difficult to visualize and cannot clearly be detected in all patients. Additionally, urinary albumin excretion was assessed with different methodologies in the various investigating centers, with this potentially leading to inaccuracy and high variability in the estimates. We cannot exclude that our results might have been biased in this sense. We used separate BP targets for nondiabetic (<140/90 mmHg) and diabetic (<130/80 mmHg) patients, which are no longer required by current hypertension guidelines [3]. However, at the time the study was planned, designed, and then started, guidelines required separate BP goals for these two populations [31]. Since the investigators adjusted treatment doses on the basis of such thresholds during the study we could not change such limits when analyzing the data, in order to avoid any possible bias on the final results. Finally, the fact that we did not observe any difference between the two treatment groups in the rate of response but we documented a significant difference in the BP drop in favor of irbesartan, even after adjustment for baseline values, might simply be the result of chance and has a limited clinical value. As a matter of fact, the primary study objective and the investigator's reference for drug titration were the BP response and not the BP reduction with treatment.

## 6. Conclusions

The present pharmacological trial demonstrated that the combination of zofenopril and hydrochlorothiazide and that of irbesartan and hydrochlorothiazide both provide similarly effective and well tolerated control of BP in hypertensive patients not responders to a previous monotherapy and with a high or very high cardiovascular risk profile. Both antihypertensive regimens effectively retarded the progression of cardiovascular, renal, and vascular damage of hypertension.

## Figures and Tables

**Table 1:** Demographic and clinical data of the patients of the intention-to-treat population at the time of randomization (*n* = 434). Data are separately shown for the two groups of randomization and reported as mean (±SD) or absolute (*n*) and relative frequency (%). BMI: Body Mass Index; LDL: Low Density Lipoprotein; HDL: High Density Lipoprotein; CV: cardiovascular; SBP: systolic blood pressure; DBP: diastolic blood pressure.

	Zofenopril 30–60 mg + HCTZ 12.5 mg	Irbesartan 150–300 mg + HCTZ 12.5 mg	*p* value
	(*n* = 213)	(*n* = 221)	
Age (years, mean ± SD)	56 ± 11	56 ± 11	0.926
Males (*n*, %)	124 (58)	120 (54)	0.411
BMI (kg/m^2^, mean ± SD)	27 ± 3	27 ± 3	0.415
Waist circumference (cm, mean ± SD)	98 ± 10	98 ± 10	0.674
Age at first diagnosis of hypertension (years, mean ± SD)	49 ± 11	50 ± 11	0.850
Concomitant diseases (*n*, %)	140 (66)	133 (60)	0.232
Alcohol drinking (*n*, %)	62 (29)	74 (33)	0.326
Cigarette smoking (*n*, %)	61 (29)	65 (29)	0.859
Diabetes (*n*, %)	23 (11)	23 (10)	0.895
Elevated total cholesterol (*n*, %)	153 (72)	165 (75)	0.506
Elevated LDL cholesterol (*n*, %)	147 (69)	156 (71)	0.721
Low HDL cholesterol (*n*, %)	51 (24)	57 (26)	0.656
Abdominal obesity (*n*, %)	181 (85)	187 (85)	0.917
Family history of premature CV disease (*n*, %)	35 (16)	32 (15)	0.574
High CV risk (*n*, %)	197 (93)	204 (92)	0.943
Sitting office SBP (mmHg)	150 ± 11	151 ± 11	0.335
Sitting office DBP (mmHg)	93 ± 7	93 ± 7	0.366

**Table 2:** Baseline-adjusted office systolic blood pressure (SBP) and diastolic blood pressure (DBP) reductions after 18 weeks of treatment with zofenopril + hydrochlorothiazide (HCTZ) or irbesartan + HCTZ in the whole study group and in the subgroup of patients treated with the low drug dose (zofenopril 30 mg or irbesartan 150 mg). Data are shown for the intention-to-treat population and reported as mean ± SD or as mean and 95% confidence interval. The *p* value refers to the statistical significance of the intertreatment difference.

Office	SBP		DBP	
	Zofenopril 30–60 mg + HCTZ 12.5 mg	Irbesartan 150–300 mg + HCTZ 12.5 mg	Zofenopril 30–60 mg + HCTZ 12.5 mg	Irbesartan 150–300 mg + HCTZ 12.5 mg
All subjects	*n* = 213	*n* = 221	*n* = 213	*n* = 221
Baseline (mmHg)	148.4 ± 9.6	149.3 ± 10.1	91.6 ± 6.7	91.6 ± 6.8
Reduction with treatment (mmHg)	15.7 (13.9, 17.5)	19.3 (17.5, 21.1)	8.6 (7.4, 9.8)	11.6 (10.4, 12.8)
*p* value	0.001		<0.001	
Low dose subgroup	*n* = 119	*n* = 104	*n* = 119	*n* = 104
Baseline (mmHg)	147.4 ± 9.5	147.4 ± 8.3	90.4 ± 6.8	91.4 ± 6.7
Reduction with treatment (mmHg)	19.5 (18.0, 21.0)	22.5 (20.7, 24.3)	12.7 (11.0, 14.4)	15.2 (13.8, 16.6)
*p* value	0.065		0.096	

**Table 3:** Summary measures for cardiac (LVMI, left ventricular mass index), renal (urine protein), and vascular (IMT, intima-media thickness) damage for the intention-to-treat population and for the two study treatment groups. For any measure the baseline value (±SD), the adjusted reduction (and 95% confidence interval), and the absolute (*n*) and relative (%) frequency of patients with damage at baseline showing regression with treatment are reported. Adjustment was made by the baseline value and other potentially confounding variables (age, gender, abdominal obesity, HDL cholesterol, family history for premature cardiovascular disease, baseline blood pressure, and blood pressure changes with treatment). The *p* value refers to the statistical significance of the intertreatment difference.

	Zofenopril 30–60 mg + HCTZ 12.5 mg	Irbesartan 150–300 mg + HCTZ 12.5 mg	*p* value
Cardiac damage	*n* = 204	*n* = 218	
Baseline LVMI (g/m^2^)	118.3 ± 35.3	124.6 ± 44.9	
LVMI reduction with treatment LVMI (g/m^2^)	7.9 (17.4, +1.5)	10.7 (19.7, 1.6)	0.467
Patients with LVH at baseline showing LVH regression at study end (*n*, %)	20/84 (23.8)	24/86 (27.9)	0.648
Renal damage			
Albumin/creatinine ratio (mg/g)	*n* = 38	*n* = 41	
Baseline	14.0 ± 23.4	10.3 ± 20.3	
Reduction with treatment	3.2 (10.3, +3.9)	5.3 (11.8, +1.2)	0.490
Microalbuminuria over the 24 hours (mg/24 h)	*n* = 56	*n* = 62	
Baseline	21.1 ± 33.0	23.2 ± 37.1	
Reduction with treatment	+0.9 (20.8, +22.6)	+8.2 (14.1, +30.5)	0.387
Semiquantitative assessment of microalbuminuria by dipstick (mg)	*n* = 87	*n* = 81	
Baseline	14.2 ± 23.4	19.3 ± 31.3	
Reduction with treatment	12.3 (20.0, 4.6)	11.6 (18.7, 4.4)	0.801
Patients with renal damage at baseline showing regression at study end (*n*, %)	9/17 (52.9)	15/22 (68.2)	0.404
Vascular damage (maximum IMT in all districts)	*n* = 211	*n* = 220	
Baseline IMT (mm)	1.22 ± 0.47	1.21 ± 0.47	—
IMT reduction with treatment (mm)	0.07 (0.15, +0.01)	0.03 (0.11, +0.05)	0.143
Patients with carotid plaque at baseline showing regression at study end (*n*, %)	18/57 (31.6)	9/56 (16.1)	0.047

**Table 4:** Rate of office blood pressure (BP) responders (<140/90 mmHg in nondiabetics and <130/80 mmHg in diabetics or high-risk patients, or SBP reduction ≥20 mmHg or DBP reduction ≥10 mmHg) and baseline-adjusted office and 24-hour SBP and DBP reductions after 48 weeks of treatment with zofenopril 60 mg + hydrochlorothiazide (HCTZ) or irbesartan 300 mg + HCTZ. Data are shown for the intention-to-treat population and reported as absolute (*n*) and relative (%) frequencies and as mean and 95% confidence interval. The *p* value refers to the statistical significance of the intertreatment difference.

	Zofenopril 60 mg + HCTZ 12.5 mg	Irbesartan 300 mg + HCTZ 12.5 mg	*p* value
Office BP responders (*n*, %)	*n* = 119	*n* = 104	
	34 (28.6)	23 (22.1)	0.178
Office BP reduction with treatment	*n* = 119	*n* = 104	
SBP (mmHg)	17.8 (15.6, 20.0)	21.2 (18.6, 23.8)	0.052
DBP (mmHg)	11.7 (10.3, 13.1)	12.9 (11.3, 14.5)	0.268
24-hour BP reduction with treatment	*n* = 50	*n* = 44	
SBP (mmHg)	7.6 (9.7, 5.6)	7.2 (9.3, 5.1)	0.744
DBP (mmHg)	4.5 (6.1, 2.8)	5.9 (7.5, 4.2)	0.250

**Table 5:** Summary measures for cardiac (LVMI, left ventricular mass index), renal (urine protein), and vascular (IMT, intima-media thickness) damage after 48 weeks of treatment with zofenopril 60 mg + hydrochlorothiazide (HCTZ) or irbesartan 300 mg + HCTZ. Data are shown for the intention-to-treat population. For any measure the baseline value (±SD), the adjusted reduction (and 95% confidence interval), and the absolute (*n*) and relative (%) frequency of patients with damage at baseline showing regression with treatment are reported. Adjustment was made by the baseline value and other potentially confounding variables (age, gender, abdominal obesity, HDL cholesterol, family history for premature cardiovascular disease, baseline blood pressure, and blood pressure changes with treatment). The *p* value refers to the statistical significance of the intertreatment difference.

	Zofenopril 60 mg + HCTZ 12.5 mg	Irbesartan 300 mg + HCTZ 12.5 mg	*p* value
Cardiac damage	*n* = 115	*n* = 104	
Baseline LVMI (g/m^2^)	118.8 ± 40.3	132.0 ± 50.3	
LVMI reduction with treatment LVMI (g/m^2^)	23.3 (40.6, 6.0)	27.0 (44.3, 9.6)	0.445
Patients with LVH at baseline showing LVH regression at study end (*n*, %)	35/44 (79.5)	39/49 (79.6)	0.989
Renal damage			
Albumin/creatinine ratio (mg/g)	*n* = 17	*n* = 12	
Baseline	12.2 ± 16.7	2.3 ± 3.6	
Reduction with treatment	+9.0 (+2.1, +15.9)	+11.7 (+3.9, +19.5)	0.348
Microalbuminuria over the 24 hours (mg/24 h)	*n* = 29	*n* = 34	
Baseline	25.5 ± 41.2	19.4 ± 32.3	
Reduction with treatment	24.8 (72.8, +23.2)	0.22 (50.6, +50.1)	0.191
Semiquantitative assessment of microalbuminuria by dipstick (mg)	*n* = 36	*n* = 27	
Baseline	10.2 ± 11.3	24.7 ± 42.9	
Reduction with treatment	6.1 (15.1, +2.9)	6.5 (15.0, +2.0)	0.924
Patients with renal damage at baseline showing regression at study end (*n*, %)	1/8 (12.5)	2/9 (22.2)	0.617
Vascular damage (maximum IMT in all districts)	*n* = 119	*n* = 104	
Baseline IMT (mm)	1.27 ± 0.53	1.23 ± 0.49	
IMT reduction with treatment (mm)	+0.24 (0.05, +0.52)	+0.28 (0.01, +0.57)	0.693
Patients with carotid plaque at baseline showing regression at study end (*n*, %)	6/32 (18.8)	8/26 (30.8)	0.190

## References

[1] Lawes C. M., Hoorn S. V., Rodgers A. (2008). Global burden of blood-pressure-related disease, 2001. *The Lancet*.

[2] Turnbull F., Blood Pressure Lowering Treatment Trialists' Collaboration (2003). Effects of different blood-pressure-lowering regimens on major cardiovascular events: results of prospectively-designed overviews of randomised trials. *The Lancet*.

[3] Mancia G., Fagard R., Narkiewicz K. (2014). 2013 ESH/ESC Guidelines for the management of arterial hypertension: the task force for the management of arterial hypertension of the European Society of Hypertension (ESH) and of the European Society of Cardiology (ESC). *Blood Pressure*.

[4] The Heart Outcome Prevention Evaluation Study Investigators (2000). Effects of an angiotensin-converting-enzyme inhibitor, ramipril, on cardiovascular events in high-risk patients. *The New England Journal of Medicine*.

[5] PROGRESS Collaborative Study Group (2001). Randomised trial of a perindopril-based blood-pressure-lowering regimen among 6105 individuals with previous stroke or transient ischaemic attack. *The Lancet*.

[6] The ALLHAT Officers and Coordinators for the ALLHAT Collaborative Research Group (2002). Major outcomes in high-risk hypertensive patients randomized to angiotensin-converting enzyme inhibitor or calcium canne blocker vs diuretic: the Antihypertensive and Lipid-Lowering treatment to prevent Heart Attack Trial (ALLHAT). *The Journal of the American Medical Association*.

[7] Dahlöf B., Sever P. S., Poulter N. R. (2005). Prevention of cardiovascular events with an antihypertensive regimen of amlodipine adding perindopril as required versus atenolol adding bendroflumethiazide as required, in the Anglo-Scandinavian Cardiac Outcomes Trial-Blood Pressure Lowering Arm (ASCOT-BPLA): a multicentre randomised controlled trial. *The Lancet*.

[8] Wald D. S., Law M., Morris J. K., Bestwick J. P., Wald N. J. (2009). Combination therapy versus monotherapy in reducing blood pressure: meta-analysis on 11,000 participants from 42 trials. *The American Journal of Medicine*.

[9] Law M. R., Wald N. J., Morris J. K., Jordan R. E. (2003). Value of low dose combination treatment with blood pressure lowering drugs: analysis of 354 randomised trials. *British Medical Journal*.

[10] Waeber B. (2003). Combination therapy with ACE inhibitors/angiotensin II receptor antagonists and diuretics in hypertension.. *Expert Review of Cardiovascular Therapy*.

[11] Ambrosioni E. (2007). Defining the role of zofenopril in the management of hypertension and ischemic heart disorders. *American Journal of Cardiovascular Drugs*.

[12] Borghi C., Bacchelli S., Esposti D. D. (2012). Long-term clinical experience with zofenopril. *Expert Review of Cardiovascular Therapy*.

[13] Borghi C., Ambrosioni E., Omboni S. (2013). Zofenopril and ramipril and acetylsalicylic acid in postmyocardial infarction patients with left ventricular systolic dysfunction: a retrospective analysis in hypertensive patients of the SMILE-4 study. *Journal of Hypertension*.

[14] Borghi C., Bacchelli S., Esposti D. D., Bignamini A., Magnani B., Ambrosioni E. (1999). Effects of the administration of an angiotensin-converting enzyme inhibitor during the acute phase of myocardial infarction in patients with arterial hypertension. *American Journal of Hypertension*.

[15] Nilsson P. (2007). Antihypertensive efficacy of zofenopril compared with atenolol in patients with mild to moderate hypertension. *Blood Pressure*.

[16] Lacourciere Y., Provencher P. (1989). Comparative effects of zofenopril and hydrochlorothiazide on office and ambulatory blood pressures in mild to moderate essential hypertension. *British Journal of Clinical Pharmacology*.

[17] Farsang C. (2007). Blood pressure control and response rates with zofenopril compared with amlodipine in hypertensive patients. *Blood Pressure*.

[18] Mallion J.-M. (2007). An evaluation of the initial and long-term antihypertensive efficacy of zofenopril compared with enalapril in mild to moderate hypertension. *Blood Pressure*.

[19] Malacco E., Piazza S., Omboni S. (2005). Zofenopril versus lisinopril in the treatment of essential hypertension in elderly patients: a randomised, double-blind, multicentre study. *Clinical Drug Investigation*.

[20] Narkiewicz K. (2007). Comparison of home and office blood pressure in hypertensive patients treated with zofenopril or losartan. *Blood Pressure*.

[21] Leonetti G., Rappelli A., Omboni S. (2006). A similar 24-h blood pressure control is obtained by zofenopril and candesartan in primary hypertensive patients. *Blood Press*.

[22] Omboni S., Malacco E., Parati G. (2009). Zofenopril plus hydrochlorothiazide fixed combination in the treatment of hypertension and associated clinical conditions. *Cardiovascular Therapeutics*.

[23] Zanchetti A., Parati G., Malacco E. (2006). Zofenopril plus hydrochlorothiazide: combination therapy for the treatment of mild to moderate hypertension. *Drugs*.

[24] Rogoza A. N., Pavlova T. S., Sergeeva M. V. (2000). Validation of A and D UA-767 device for the self-measurement of blood pressure. *Blood Pressure Monitoring*.

[25] Palatini P., Frigo G., Bertolo O., Roman E., Da Cortà R., Winnicki M. (1998). Validation of the A&D TM-2430 device for ambulatory blood pressure monitoring and evaluation of performance according to subjects' characteristics. *Blood Pressure Monitoring*.

[26] http://www.morepress.net/.

[27] Reeves S. T., Glas K. E., Eltzschig H. (2007). Guidelines for performing a comprehensive epicardial echocardiography examination: recommendations of the American Society of Echocardiography and the Society of Cardiovascular Anesthesiologists. *Journal of the American Society of Echocardiography*.

[28] Devereux R. B., Casale P. N., Kligfield P. (1986). Performance of primary and derived M-mode echocardiographic measurements for detection of left ventricular hypertrophy in necropsied subjects and in patients with systemic hypertension, mitral regurgitation and dilated cardiomyopathy. *The American Journal of Cardiology*.

[29] Du Bois D., Du Bois E. F. (1989). A formula to estimate the approximate surface area if height and weight be known. 1916. *Nutrition*.

[30] Stein J. H., Korcarz C. E., Post W. S. (2009). Use of carotid ultrasound to identify subclinical vascular disease and evaluate cardiovascular disease risk: summary and discussion of the american society of echocardiography consensus statement. *Preventive Cardiology*.

[31] Mancia G., De Backer G., Dominiczak A. (2007). 2007 guidelines for the management of arterial hypertension: the task force for the management of arterial hypertension of the European Society of Hypertension (ESH) and of the European Society of Cardiology (ESC). *Journal of Hypertension*.

[32] Parati G., Omboni S., Malacco E. (2006). Antihypertensive efficacy of zofenopril and hydrochlorothiazide combination on ambulatory blood pressure. *Blood Pressure*.

[33] Bramlage P. (2009). Fixed combination of irbesartan and hydrochlorothiazide in the management of hypertension. *Vascular Health and Risk Management*.

[34] Agabiti-Rosei E., Manolis A., Zava D., Omboni S. (2014). Zofenopril plus hydrochlorothiazide and irbesartan plus hydrochlorothiazide in previously treated and uncontrolled diabetic and non-diabetic essential hypertensive patients. *Advances in Therapy*.

[35] Parati G., Omboni S., Palatini P. (2008). Italian society of hypertension guidelines for conventional and automated blood pressure measurement in the office, at home and over 24 hours. *High Blood Pressure & Cardiovascular Prevention*.

[36] Sica D. A. (2002). Rationale for fixed-dose combinations in the treatment of hypertension: the cycle repeats. *Drugs*.

[37] Schrader J., Bramlage P., Lüders S., Thoenes M., Schirmer A., Paar D. W. (2007). BP goal achievement in patients with uncontrolled hypertension: results of the treat-to-target post-marketing survey with irbesartan. *Clinical Drug Investigation*.

[38] Sun N.-L., Jing S., Chen J. (2005). The control rate of irbesartan/hydrochlorothiazide combination regimen in the treatment of Chinese patients with mild to moderate hypertension. *Zhonghua Xin Xue Guan Bing Za Zhi*.

[39] Bobrie G., Delonca J., Moulin C., Giacomino A., Postel-Vinay N., Asmar R. (2005). A home blood pressure monitoring study comparing the antihypertensive efficacy of two angiotensin II receptor antagonist fixed combinations. *American Journal of Hypertension*.

[40] Neutel J. M., Saunders E., Bakris G. L. (2005). The efficacy and safety of low- and high-dose fixed combinations of irbesartan/hydrochlorothiazide in patients with uncontrolled systolic blood pressure on monotherapy: the INCLUSIVE trial. *Journal of Clinical Hypertension*.

[41] Coca A., Calvo C., García-Puig J. (2002). A multicenter, randomized, double-blind comparison of the efficacy and safety of irbesartan and enalapril in adults with mild to moderate essential hypertension, as assessed by ambulatory blood pressure monitoring: the MAPAVEL study. *Clinical Therapeutics*.

[42] Lacourcière Y. (2000). A multicenter, randomized, double-blind study of the antihypertensive efficacy and tolerability of irbesartan in patients aged ≥65 years with mild to moderate hypertension. *Clinical Therapeutics*.

[43] Chiou K. R., Chen C. H., Ding P. Y. (2000). Randomized, double-blind comparison of irbesartan and enalapril for treatment of mild to moderate hypertension. *Zhonghua Yi Xue Za Zhi*.

[44] Mimran A., Ruilope L., Kerwin L. (1998). A randomised, double-blind comparison of the angiotensin II receptor antagonist, irbesartan, with the full dose range of enalapril for the treatment of mild-to-moderate hypertension. *Journal of Human Hypertension*.

[45] Angulo E., Robles N. R., Grois J., Barquero A., Pérez Miranda M. (2002). Comparison of the antihypertensive activity of fosinopril and irbesartan. *Anales de Medicina Interna*.

[46] Kunz R., Friedrich C., Wolbers M., Mann J. F. E. (2008). Meta-analysis: effect of monotherapy and combination therapy with inhibitors of the renin-angiotensin system on proteinuria in renal disease. *Annals of Internal Medicine*.

[47] Forni V., Wuerzner G., Pruijm M., Burnier M. (2011). Long-term use and tolerability of irbesartan for control of hypertension. *Integrated Blood Pressure Control*.

